# Clinical Clues of Pre-Symptomatic Pancreatic Ductal Adenocarcinoma Prior to Its Diagnosis: A Retrospective Review of CT Scans and Laboratory Tests

**DOI:** 10.3390/clinpract12010008

**Published:** 2022-01-17

**Authors:** Hwe Hoon Chung, Kyung Sook Lim, Joo Kyung Park

**Affiliations:** 1 Samsung Medical Center, Department of Medicine, Sungkyunkwan University School of Medicine, Suwon 06351, Korea; hwehoon@hotmail.com (H.H.C.); kyungsuk2722@hanmail.net (K.S.L.); 2 Department of Health Sciences and Technology, Samsung Advanced Institute for Health Sciences & Technology, Sungkyunkwan University, Seoul 06355, Korea

**Keywords:** pancreatic neoplasms, diagnosis, diabetes mellitus

## Abstract

Most pancreatic ductal adenocarcinoma cases are unresectable at the time of diagnosis. Only early diagnosis and curative resection can help prolong survival. We tried to find out useful clinical clues of pre-symptomatic area prior to pancreatic cancer diagnosis compared to normal controls. Of 4799 patients diagnosed with pancreatic cancer between 1995 and 2014 at the Samsung Medical Center, 51 were selected for study. They had no symptoms at diagnosis and underwent computed tomography 6 to 36 months prior to diagnosis for reasons other than cancer diagnosis. We selected 288 control subjects who underwent computed tomography during the same period. Data were retrospectively reviewed included various variables. Fasting blood sugar (171.8 ± 97.5 vs. 115.8 ± 34.8 units, *p* < 0.05), new onset diabetes mellitus within 3 years (12/51 (23.5%) vs. 17/181 (9.8%), *p* < 0.05), carbohydrate antigen 19-9 level (609.5 ± 2342.5 vs. 17.0 ± 26.2, *p* = 0.08), main pancreatic duct dilatation (26/51 (51.0%) vs. 57/181 (31.5%), *p* < 0.05) in computed tomography scan were higher in pancreatic cancer group than in normal group, respectively. In multi-variate analysis, carbohydrate antigen 19-9, new onset diabetes mellitus (<3 years), and segmental main pancreatic duct dilatation were independent risk factors for pancreatic cancer. Our study concluded that independent risk factors for pancreatic cancer were elevated carbohydrate antigen 19-9, new onset diabetes mellitus (<3 years), and local main pancreatic ductal dilatation on computed tomography scan.

## 1. Introduction

Pancreatic ductal adenocarcinoma (PDAC) has a low incidence but is frequently fatal. The dismal prognosis is mainly because 80–90% of patients have unresectable disease at the time of disease diagnosis [1].

The association between diabetes mellitus (DM) and PDAC has long been recognized. While long-standing DM is thought to be an etiologic factor for pancreatic cancer, new-onset DM may be a manifestation of the cancer. Up to 85% of patients with pancreatic cancer have DM or hyperglycemia, which frequently manifests as early as 2–3 years before a diagnosis of pancreatic cancer [2]. Patients with new-onset DM have 5- to 8-fold increased risk of being diagnosed with pancreatic cancer within 1–3 years of developing diabetes. In one study, the prevalence of DM in pancreatic cancer patients was reported to be 40%, and half of the DM patients with pancreatic cancer had new-onset DM with a duration of 2 years or less [3]. The relationship of new-onset DM and pancreatic cancer remains unknown. One possible explanation for the high prevalence of DM in pancreatic cancer is that DM is simply a consequence of glandular destruction by the tumor and so is a late manifestation of the cancer. Two studies have prospectively screened for pancreatic cancer in new-onset diabetes using symptoms and carbohydrate antigen 19-9 (CA 19-9) elevation as secondary sieves. Although the prevalence of pancreatic cancer in the screened population was high (4.7 and 13%), most identified cancers were unresectable, again reiterating that screening for PDAC will have to be performed in asymptomatic subjects [4,5].

Previous reports demonstrated that the presence of focal hypo-attenuation and pancreatic duct dilatation on pre-diagnostic computed tomography (CT) scans are useful findings for the early diagnosis of pancreatic cancer. Main pancreatic duct (MPD) interruption and dilatation were early findings of pancreatic cancer on pre-diagnostic CT scans [6,7].

Only early diagnosis and curative resection can prolong survival. Although many attempts for early diagnosis have been tried, early signs of PDACs are not fully identified. Clinical clues of pre-symptomatic pancreatic cancer diagnosis should be further investigated and may give provide insights to the early diagnosis of pancreatic cancer.

We tried to find out useful clinical clues of pre-radiologic and pre-symptomatic area prior to pancreatic cancer diagnosis patients compared to normal controls.

## 2. Materials and Methods

### 2.1. Study Design

The study was approved by the Samsung Medical Center Institutional Review Board. The retrospective case-control study involved patients with pancreatic cancer diagnosis from September 1995 to December 2014 and who were found incidentally without symptoms. Electronic medical records were reviewed with clinical features and all CT scans were blindly reviewed by professional radiologist.

Of these patients, we selected patients who underwent CT scan prior to the pancreatic cancer diagnosis from April 1997 to June 2014. Furthermore, we excluded some patients because of not available CT scan data, limited evaluation due to artifact, and previous pancreatic mass. Finally, these patients were selected as PDAC groups.

**Figure 1:**
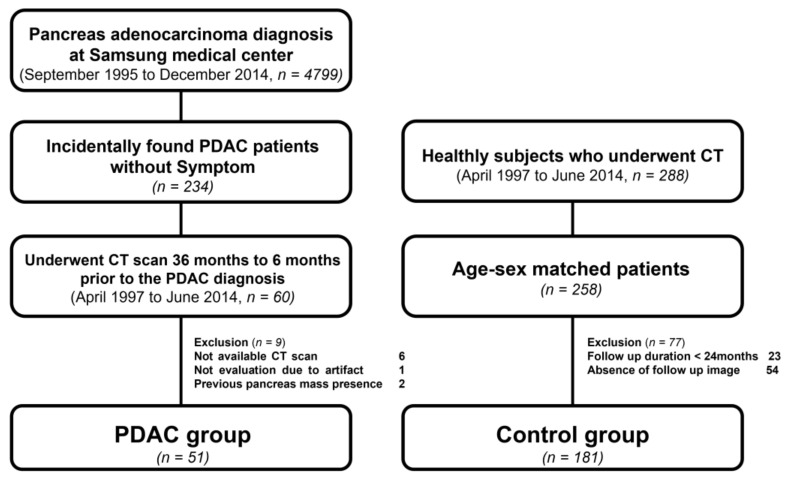
Study design and patients enrollment.

Healthy subjects performed CT scans from April 1997 to June 2014 for other than pancreatic cancer diagnosis. For healthy subjects, age and gender were roughly matched to the patient group. Of these, we excluded subjects because of follow-up image within 24 months or lack of follow-up images. Finally, we selected these subjects as the control group (Figure 1).

Data that were retrospectively reviewed included age; gender; levels of CA 19-9, carcinoembryonic antigen (CEA), fasting blood sugar and glycated hemoglobin (HbA1C) at diagnostic stage; DM; DM onset, impaired fasting blood glucose (IFG) and CT scans prior to pancreatic cancer diagnosis. History of smoking, chronic pancreatitis and pancreatic cancer family history were also reviewed. CT images were studied for the presence of subtle density change and presence of main pancreatic duct dilatation and type. Presence and type of cyst, and accompanying acute pancreatitis were also blindly reviewed by the radiologist. DM was judged to be present if the patient had a FBS ≥ 126 mg/dL or was receiving oral hypoglycemic agents and/or insulin. Date of onset of DM was defined as the date of the first FBS ≥ 126 mg/dL with a prior normal FBS. Study and control patients are roughly matched, so that the range of age distribution was identical between cases and controls within each gender group. Possible unbalance in distribution of predictors was adjusted through multivariate analysis. Patients received CT scans at the same times as study patients. Control patients with pancreatic cancer diagnosis were still alive through at least 24 months. The study patients were evaluated for clinical changes and CT scans 36, 24, 12 and 6 months prior to pancreatic cancer diagnosis.

### 2.2. Study Patients

**Figure 2:**
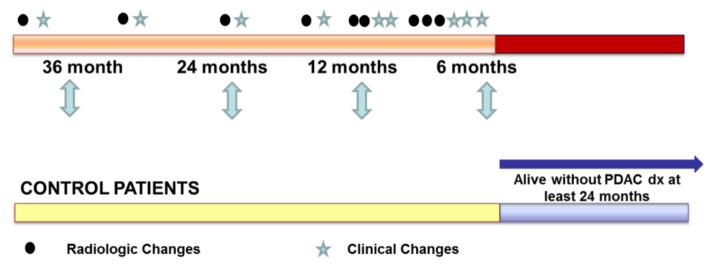
Study flow of clinical and radiologic evaluation.

A total of 4799 patients were investigated for PDAC diagnosis from September 1995 to December 2014 at Samsung Medical Center. A total of 234 asymptomatic patients were incidentally discovered to have PDAC. Further, 60 patients underwent CT scans 36–6 months prior to the PDAC diagnosis from April 1997 to June 2014 for reasons other than cancer diagnosis. Of these, 9 patients were excluded; CT data were unavailable for 6, one case could not evaluate due to an artifact, and 2 cases displayed a previous pancreatic mass. Further, 51 patients were selected for the PDAC group. A total of 288 healthy subjects underwent CT scan from April 1997 to June 2014. Age and sex were matched for 258 patients. Further, 77 patients were excluded; 23 patients had a follow-up duration <24 months and 54 patients did not have follow-up image data. In addition, 181 healthy subjects were selected for the control group (Figure 1). Study patients received a CT scan and clinical changes were evaluated 36, 24 and 12 months prior to diagnosis; some patients received two scans at 12 months and all patients received three CT scans and evaluations 6 months prior to PDAC diagnosis. Control patients received CT scans and evaluations of clinical changes over the same period. Control patients were alive without PDAC diagnosis at least 24 months after study patients had been diagnosed as PDAC (Figure 2).

### 2.3. Statistical Analyses

Descriptive statistics are presented as mean and standard deviation (SD). Independent sample Student’s *t*-tests were computed for continuous variables. Chi-squared analysis with calculation of OR was performed for categorical variables. Logistic regression analysis was used to identify risk factors of PDAC. Multi-variate analysis was performed on variables that were associated with PDAC occurrence on univariate analysis (*p* < 0.200). Statistical significance was defined as *p* < 0.05.

## 3. Results

Fifty-one patients with pancreatic cancer and one hundred eighty-one normal patients are enrolled. PDAC group and the normal group were compared for clinical characteristics and CT scan findings (Table 1). Normal subjects with CT scan data were roughly matched with age and sex during the same period of follow ups in PDAC group.

FBS was higher level in the PDAC group (171.8 ± 97.5 units) than the normal group (115.8 ± 34.8 units) (*p* < 0.05). However, there was no statistical significance concerning total bilirubin, HbA1C, CEA, CA 19-9 level between the two groups.

DM onset before 3 years occurred in 9 (17.6%) PDAC patients, 39 (22.4%) normal subjects, and DM onset sooner than 3 years evident in 12 (23.5%) of PDAC patients and 17 (9.8%) normal subjects (*p* < 0.05). Accompanying chronic pancreatitis, impaired fasting glucose, DM, smoking and family history of PDAC was not showed the significant difference between two groups.

**Figure 3:**
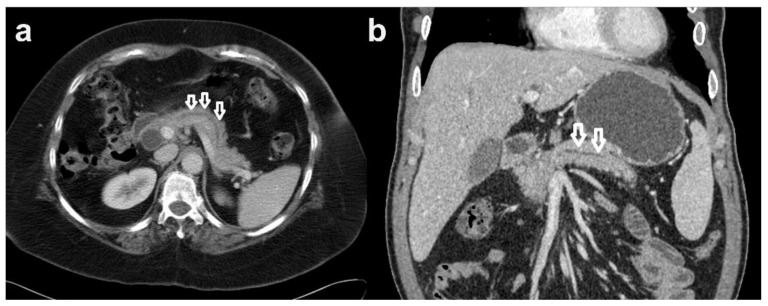
Pancreatic duct dilatation on CT scan. (**a**) CT scan - axial view of pancreatic duct dilatation. (**b**) CT scan - coronal view of pancreatic duct dilatation.

A total of 51 patients were performed CT scan prior to pancreatic cancer diagnosis. Presence of main pancreatic duct dilatation was higher in the PDAC group (51.0%) than normal control group (31.5%) (*p* < 0.05).*(Figure 3) Presence of subtle density changes, pancreas cyst and coexistence of acute pancreatitis showed meaningless difference between two groups.

CT scans revealed tumors in the head (*n* = 26, 51.0%), body and tail (*n* = 11, 21.6%) and tail (*n* = 14, 27.5%). Tumor stage was Ia + Ib (*n* = 4, 7.8%), IIa (*n* = 16, 31.4%), IIb (*n* = 14, 27.5%), III (*n* = 3, 5.9%) and IV (*n* = 14, 27.5%). The time between CT scan and diagnosis of PDAC was 15.96 ± 7.95 months. Furthermore, overall survival was 21.4 ± 20.8 months in PDAC group (Table 2). Multivariate analysis showed that CA 19-9, new onset DM (<3 years), and segmental main pancreatic duct dilatation were independent risk factors for PDAC (Table 3).

## 4. Discussion

Most pancreatic cancer patients are in the advanced stage of the disease at presentation. The 5-year survival rate of pancreatic cancer is <5% and the prognosis is poor. It is because diagnosis of pancreatic cancer is often diagnosed lately due to the vague gastrointestinal symptoms. Our study showed that for the diagnosis of resectable pancreatic cancer, new onset DM (<3 years), CA 19-9 and CT scan are predictive diagnostic clues in pre-symptomatic state of pancreatic cancer. Resectable pancreatic cancer refers to a lesion capable of R0 resection because it does not invade major blood vessels or have distant metastasis.

For detection of resectable pancreatic cancer, additional evaluation needs to target patients who have predictive diagnostic clue.

We found an association between new-onset DM within 3 months and pre-symptomatic PDAC. The diagnosis of DM is based on the American Diabetes Association criteria of FBS ≥ 126 mg/dL [8]. FBS was 171.8 ± 97.5 mg/dL in the PDCA group and 115.8 ± 34.8 mg/dL in the normal group. DM onset within 3 years was evident in 23.5% of PDAC patients (12/51) and 9.8% of healthy patients (17/181). DM onset within 3 years was revealed as a predictive risk factor for pre-symptomatic PDAC diagnosis. Previous studies suggested new-onset hyperglycemia and diabetes are the only significant clues to the presence of sporadic PDAC before developing cancer-related symptoms. Numerous studies have suggested that new-onset diabetes is present in nearly half of patients and is associated with the early stage of all PDAC cases. The historical prevalence of DM in PDAC varies from 4 to 64% depending on applied criteria to identify or classify DM patient [9]. In a recent study, we found that the cancer is often resectable at the onset of DM [10]. Recent reports have emphasized new-onset DM as an early manifestation of PDAC [3,9]. However, it is neither cost-effective nor practical to screen all patients with new-onset DM because the prevalence of PDAC in new-onset DM is less than 1% [11,12,13].

Nevertheless, compared to the general population, patients with new onset DM are eight times more likely to have PDAC [11]. Thus, in new onset DM patients with other risk factor of PDAC, the possibility of early PDAC may be considered.

Presently, CT scan abnormalities of local MPD dilatation were identified as a risk factor to identify pre-symptomatic PDAC. The presence of focal hypo-attenuation and pancreatic duct dilatation on pre-diagnostic CT exams are useful findings for the early diagnosis of PDAC.

In our study, resectable stage of PDAC was evident in 51.0% of the patients (26/51) over 6 months before PDAC diagnosis. The retrospectively review of 114 CT scans at or before cancer diagnosis revealed that PDAC was either undetectable or resectable on scans over 6 months before clinical diagnosis. On the other hand, other authors have described that when the diagnosis was made 6 months before the onset of the symptoms, many cases of operable radiologic findings are found [7,10]. These studies suggest that PDAC is resectable as little as 6 months before clinical diagnosis, when it is still asymptomatic and would not normally come to clinical attention [14]. Further, in future studies, we will be able to take a closer approach to finding resectable pancreatic cancer in clinical practice through comparative studies on the characteristics of resectable vs unresectable pancreatic cancer.

Presently, CA 19-9 was a predictive risk factor prior to diagnosis of PDAC diagnosis, as has been described. The sensitivity, specificity, and positive predictive value of CA 19-9 are very high in diagnosing PDAC in patients suspected of pancreatic diseases [15,16,17,18,19,20,21]. In contrast to these CA 19-9 predictive risk factor, several studies reported that screening test of CA 19-9 was not a useful predictive factor for asymptomatic PDAC patient diagnosis. Several attempts at screening for PDAC using changes in CA 19-9 levels as an early predictive factor of PDAC have been reported, but they had limited success [22,23,24]. The CA 19-9 level is elevated in 87% of PDAC patients, and serum CA 19-9 concentration is highly correlated to the tumor size in most, if not in all patients with PDAC [25,26]. Mass screening for PDAC using CA 19-9 levels in asymptomatic subjects is ineffective because of a very low positive predictive value, despite its high sensitivity and specificity [23].

The limitation of our study was its retrospective nature. As a pilot study for a future prospective study, it was difficult to find exact cases and to include relevant controls. In the selection of patient group and control group, it was not accurately matched due to the limitations of retrospective studies. We roughly matched with age and gender to compensate for this, but it could still be a limitation. Advantages of our study include the age- and sex-matches, blind review of CT scans by radiologist and minimal bias in selecting the control group. Univariate analysis results showed advanced age, male sex, elevated CA 19-9, new-onset DM within 3 years, presence of subtle density change on CT scan and presence of local dilatation on CT scan as influential factors. Multivariate analysis result showed elevated CA 19-9, new-onset DM within 3 years and local MPD dilatation on CT scan were influential.

Based on this, we recommend that patients who visited the hospital with asymptomatic or other symptoms undergo follow-up test with the risk of developing pancreatic cancer if elevated CA 19-9 and new-onset DM within 3 years and local MPD dilation on CT were observed.

## 5. Conclusions

We concluded that carbohydrate antigen 19-9, new onset diabetes mellitus (<3 years), and local main pancreatic ductal dilatation on computed tomography scan were found to be independent factors of pancreatic cancer.

## Figures and Tables

**Table 1:** Baseline characteristics in all study subjects.

Characteristics	PDAC Group (*n* = 51)	Normal Group (*n* = 181)	*p*-Value
Age, years	66.1 ± 9.8	61.5 ± 8.8	0.195
Gender			0.213
Male	32	112	
Female	19	69	
Total bilirubin (mg/dL)	0.8 ± 0.6	0.6 ± 0.3	0.060
FBS (mg/dL)	171.8 ± 97.5	115.8 ± 34.8	0.000
HbA1C (%)	7.7 ± 2.3	6.7 ± 1.6	0.086
CEA (ng/mL)	13.8 ± 70.7	1.8 ± 1.2	0.294
CA 19-9 (U/mL)	609.5 ± 2342.5	17.0 ± 26.2	0.083
Chronic pancreatitis (%)	8 (15.7)	42 (23.2)	0.249
IFG (%)	5 (17.9)	23 (12.7)	0.574
DM (%)	21 (41.2)	63 (34.8)	0.403
DM onset (%)	21 (41.2)	56 (30.9)	0.035
>3 years (%)	9 (17.6)	39 (22.4)	
<3 years (%)	12 (23.5)	17 (9.8)	
Smoking (%)	15 (29.4)	60 (33.1)	0.614
Smoking (PY)	7.5 ± 13.9	8.1 ± 14.3	0.318
PDAC FHx (%)	1 (2.0)	12 (6.6)	0.200
Presence of subtle density changes on CT			0.005
High-density (%)	1 (2.0)	1 (0.6)	0.244
Low-density (%)	2 (3.9)	2 (1.1)	
Iso-density (%)	2 (3.9)	0 (0.0)	
Presence of MPD dilatation on CT (%)	26 (51.0)	57 (31.5)	0.000
Segmental (%)	15 (29.4)	9 (5.0)	
Diffuse (%)	11 (21.6)	46 (25.4)	
Presence of cyst (%)	19 (37.3)	64 (35.4)	0.000
Single (%)	5 (10.0)	32 (17.7)	
Multiple (%)	6 (12.0)	11 (6.1)	
IPMN-like (%)	8 (16.0)	21 (11.6)	
Acute pancreatitis (%)	2 (4.2)	3 (1.7)	0.290

FBS: fasting blood sugar, HbA1C: hemoglobin A1c, CEA: carcinoembryonic antigen, CA 19-9: carbohydrate antigen 19-9, IFG: impaired fasting glucose, FHx: family history, MPD: main pancreatic duct, IPMN: intrapancreatic mucinous neoplasm.

**Table 2:** Characteristics of PDAC group.

	PDAC Group (*n* = 51)
Tumor location	
Head	26 (51.0)
Body, Body + tail	11 (21.6)
Tail	14 (27.5)
Stage	
Ia + Ib	4 (7.8)
IIa	16 (31.4)
IIb	14 (27.5)
III	3 (5.9)
IV	14 (27.5)
Time interval between CT scan and the diagnosis of PDAC (months)	15.96 ± 7.95
Overall survival (months)	21.4 ± 20.8

PDAC: pancreatic ductal adenocarcinoma.

**Table 3:** Risk factors associated with PDAC.

Variables	Univariate		Multivariate	
	OR (95% CI)	*p* -Value	OR (95% CI )	*p*-Value
Age (year)	1.06 (1.02–1.10)	0.195	1.04 (0.99–1.09)	0.122
Gender	1.21 (0.53–2.31)	0.213	1.15 (0.43–3.07)	0.788
Female				
Male				
CA 19-9 (U/mL)	1.02 (1.01–1.04)	0.000	1.02 (1.01–1.03)	0.000
Chronic pancreatitis	0.62 (0.27–1.41)	0.252		
DM and IFG				
No	1	0.666		
IFG	0.83 (0.29–2.39)	0.725		
DM	1.27 (0.65–2.46)	0.484		
DM onset				
No	1	0.043		0.038
>3 years	0.91 (0.40–2.08)	0.819	0.81 (0.24–2.75)	0.735
<3 years	2.78 (1.20–6.44)	0.017	4.60 (1.32–16.06)	0.017
Smoking	0.84 (0.43-1.65)	0.614		
Family history	0.28 (0.04–2.22)	0.229		
Presence of subtle density change	6.45 (1.49–27.98)	0.013	1.10 (0.08–15.25)	0.942
MPD dilatation	1	0.000		0.019
Segmental	8.40 (3.31–21.3)	0.000	6.68 (1.76–25.41)	0.005
Diffuse	1.21 (0.55–2.64)	0.641	1.22 (0.41–3.65)	0.718

CA 19-9: carbohydrate antigen 19-9, IFG: (impaired fasting glucose), MPD: main pancreatic duct.

## Data Availability

Not applicable.
